# Audiological Outcome of the Simultaneous Tumor Resection and Cochlear Implantation in Two Cases of Sporadic and Two Cases of Neurofibromatosis Type 2-Associated Intracochlear Schwannoma

**DOI:** 10.3390/jcm13113042

**Published:** 2024-05-22

**Authors:** Abdullah A. AlMutawah, Taegyeong Kim, Jong Woo Chung

**Affiliations:** 1 Department of Otorhinolaryngology-Head and Neck Surgery, Asan Medical Center, University of Ulsan College of Medicine, Seoul 05505, Republic of Korea; aalmutawah@gmail.com (A.A.A.); tgkim87@gmail.com (T.K.); 2 Department of Otorhinolaryngology-Head and Neck Surgery, Jahra Hospital, Ministry of Health, Jahra 00043, Kuwait

**Keywords:** vestibular schwannoma, hearing loss, cochlear implantation, rehabilitation, case report

## Abstract

**Objectives**: Simultaneous removal and cochlear implantation (CI) have been reported in intralabyrinthine and intracochlear schwannoma. A wide range of postoperative hearing outcomes have been reported after CI in these cases. This study evaluated the outcomes of performing a simultaneous resection of Schwannoma in cochlea and cochlear implantation (CI), aiming to assess the effectiveness of this combined surgical approach for hearing rehabilitation with CI. **Methods**: This retrospective case series was conducted at a tertiary care center. The study included four consecutive patients with profound sensorineural hearing loss due to a mass inside the cochlea. These patients underwent simultaneous single-sided CI and tumor resection performed by the same surgeon. Preoperative and postoperative audiological assessments were conducted to evaluate the patients’ hearing outcomes before and after the surgical intervention. **Results**: Simultaneous CI with tumor resection was successful in all cases. Two of the four patients had a unilateral tumor, while the other two had a bilateral tumor with the involvement of the internal auditory canal and cerebellopontine angle (neurofibromatosis type 2 (NF2)). In two cases of unilateral tumor, aided free-field pure tone average (PTA) was 26 dB, and 46 dB hearing level (HL), and word recognition score (WRS) at 65 dB was 40% and 68%, respectively, 3 months after surgery. In two cases of tumor with NF2, aided free-field PTA was 36 dB and 60 dB HL, and both cases showed 0% WRS at 65 dB 3 months after surgery. **Conclusions**: Simultaneous schwannoma excision and CI in patients with Schwannoma inside cochlea are surgically practical and safe. Postoperatively, there was a notable improvement in hearing in cases of sporadic schwannoma, regardless of the type of CI used. However, there was 0% WRS in the two NF2 patients with a mass in the internal auditory canal.

## 1. Introduction

Vestibular Schwannoma (VS) is a slow-growing, benign tumor that arises from Schwann cells of the eighth cranial nerve. It primarily originates from the inferior division of the vestibular nerve, though it can also arise from the superior vestibular division or the cochlear nerve [1]. VS can manifest unilaterally, predominantly in sporadic cases, or bilaterally when associated with neurofibromatosis type 2 (NF2), which is less common [2]. It is the third most prevalent nonmalignant intracranial tumor, following meningiomas and pituitary adenomas [3].

Intralabyrinthine schwannoma (ILS) is a specific subtype of vestibular schwannoma that originates exclusively in the membranous labyrinth from peripheral branches of the cochlear or vestibular nerves [4]. The enhanced capabilities of modern imaging modalities have increased the diagnosis rate of ILS, whereas, previously, the incidence was 0.81 per 100,000 persons–year [5]. Most documented cases of ILS are located in the intra-cochlear area [6].

ILS is rare but should be differentiated from cochleovestibular disorders such as Meniere’s disease and vestibular neuritis [7]. It may lead to various symptoms, including unilateral hearing loss, tinnitus, vertigo or dizziness, and ear fullness.

Imaging studies, particularly magnetic resonance imaging (MRI) and computed tomography (CT), play a critical role in confirming the diagnosis of ILS and assessing the extent of the tumor. MRI is considered the gold standard for detecting ILS. MRI reveals characteristic features of ILS, such as contrast enhancement on T1-weighted images and filling defects on T2-weighted images [8]. These findings are essential for accurately differentiating ILS from other conditions, aiding in surgical planning and follow-up [7]. For hearing rehabilitation, simultaneous mass removal and CI have recently been used. Postoperative satisfactory hearing outcomes have been reported in these patients [9,10,11]. However, masses involving a wide range of cochlea and IAC may yield worse results because neural dysfunction could be expected [12]. The various management methods for ILS include observation (watchful waiting), surgery, and radiotherapy [13]. The issue of managing single-sided deafness (SSD) as a sequel to the disease or its treatment remains. Hearing rehabilitation through cochlear implants (CI), either simultaneously with tumor excision or as a second-stage surgery, or insertion through schwannoma, is an evolving part of the treatment of ILS [9,12,14].

In this study, we aimed to describe four cases of Schwannoma inside the cochlea or vestibule where a concurrent CI was conducted during the tumor excision procedure and, subsequently, to evaluate the audiological outcome.

## 2. Methods

The study is a retrospective case series conducted at a tertiary care center. It included four consecutive patients with profound sensorineural hearing loss caused by a mass inside the cochlea, who underwent single-sided CI with simultaneous tumor removal performed by the same surgeon. The protocol for collecting patient data was approved by the Institutional Review Board of Asan Medical Center (2023-1374), and the requirement for obtaining informed consent forms was waived.

Pure-tone audiometry (PTA) was used to evaluate the hearing levels at frequencies ranging from 0.5 to 8 kHz. The word recognition score (WRS) for monosyllabic stimuli at 60 dB was also evaluated, with complete masking of the contralateral ear. Follow-up evaluations were conducted at 3 and 6 months, with further assessments carried out 1 and 2 years after surgery.

An MRI of the temporal bone was performed for the evaluation of a schwannoma.

## 3. Results

### 3.1. Case 1

**Figure 1:**
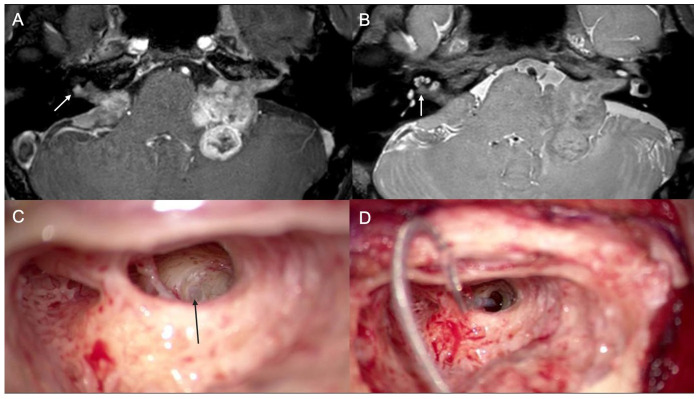
Case #1. Preoperative T1-weighted MR with gadolinium enhancement (**A**) and proton density-weighted MR image (**B**) show a small mass in the basal turn of the cochlea (white arrows). Through the round window, a soft tissue mass is shown (black arrow) (**C**). After removal of the mass, the electrode of the cochlear implant is introduced and fully inserted (**D**).

A 41-year-old male reported bilateral progressive hearing loss for 2 years on the left side and 20 years on the right side. Temporal bone MRIs showed bilateral vestibular schwannomas. (Figure 1) There were extensive soft tissue masses involving both CPA, IAC, and the right cochlear basal turn. A mutation of the NF-2 gene was found in the genetic study. The preoperative right-side aided (hearing aid) speech detection threshold (SDT) was 62 dB HL with 0% WRS at 65 dB HL. The left side was deaf. A mass excision was performed on the left side via a suboccipital approach. Stereotactic radiosurgery was applied to the mass on the right side. Four months after the stereotactic radiosurgery, CI was performed on the right side. During surgery, a mass was found in the basal turn of the cochlea (Figure 1). The mass could be removed en bloc. After mass removal, the cochlear basal turn was patent, and the CI electrode could be inserted without resistance. Three months later, the right-side aided PTA was 36 dB HL in the free field, but the WRS remained at 0%. The CI device used was the AB Midscala from Advanced Bionics.

### 3.2. Case 2

**Figure 2:**
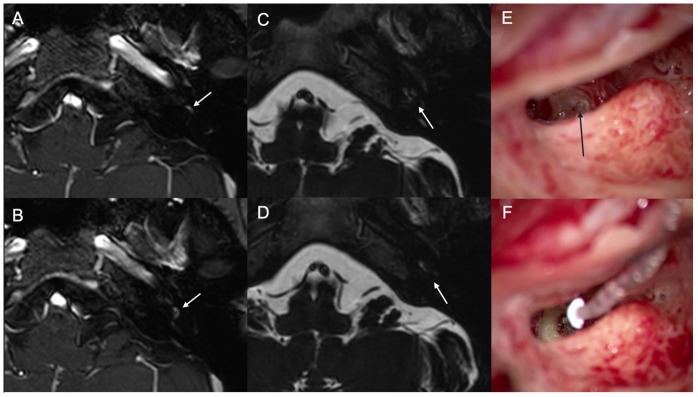
Case #2 (**A**–**F**). Preoperative T1-weighted MR with gadolinium enhancement shows the enhancing mass in the basal turn of the cochlea (**A**) and vestibule (**B**) (white arrows). T2-weighted MR image shows a filling defect in the basal turn of the cochlea (**C**) and vestibule (**D**) (white arrows). Through the round window, a soft tissue mass is shown (black arrow) (**E**). After removal of the mass, the electrode of the cochlear implant is introduced and fully inserted (**F**).

A 62-year-old female was found to have an intralabyrinthine schwannoma. She reported progressive hearing loss and tinnitus in her left ear. The size of the intralabyrinthine mass remained stable for 10 years, with no extension into the internal auditory canal. However, she continued to experience hearing loss and intractable tinnitus on the left side. Preoperatively, the left side was deaf. WRS was 0%. MR image showed a mass in the vestibule with extension to cochlear basal turn (Figure 2). Cochlear implantation was performed on the left side. During surgery, the sclerotic basal turn of the cochlea was identified, but cochleostomy was successfully performed. The mass in the basal turn of cochlea was removed (Figure 2). The electrode was inserted to the level of the round window. The cochleostomy site was sealed with harvested fascia and muscle. Three months after the cochlear implant, the left-side aided PTA in the free field was 26 dB, and the word recognition score was 40% at 65 dB 3 months after the CI. The CI device used was Nucleus CI632 from Cochlear.

### 3.3. Case 3

**Figure 3:**
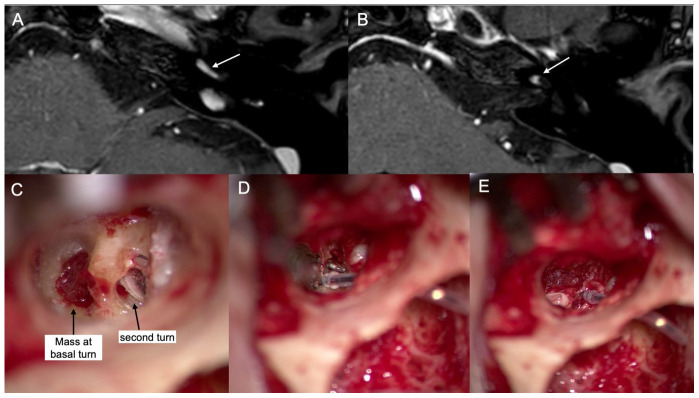
Case #3 (**A**–**E**). Preoperative T1-weighted MR with gadolinium enhancement shows the enhancing mass in the basal turn (**A**) and second turn (**B**) of the cochlea (white arrows). Operative findings (**C**–**E**): mass at the basal turn and second turn of the cochlea (black arrows) (**C**), electrode in the basal and second turns (**D**), and repair of the cochlea with fascia and cartilage (**E**).

A 52-year-old female reported left-sided hearing disturbance and tinnitus, which started 2 years ago. The initial pure tone audiometry showed left-side sensorineural hearing loss, and the speech audiometry revealed an 8% word recognition score at 100 dB. This led to a temporal bone MR that identified an intracochlear schwannoma. The intracochlear mass was identified at the basal turn and second turn of the cochlea (Figure 3). To expose the mass in the basal and second turn, the lateral bony wall of the cochlea was partially removed via transcanal approach. After removal of the mass from the basal half of the second turn, the cochlear lumen was patent. The CI electrode could be inserted without resistance. The opened cochlear bony wall was reconstructed using cartilage and fascia. Three months after the CI, the left-side aided PTA was 46 dB, and the WRS was 68% at 65 dB. The CI device used was the Neuro ZtiEVO from Oticon.

### 3.4. Case 4

**Figure 4:**
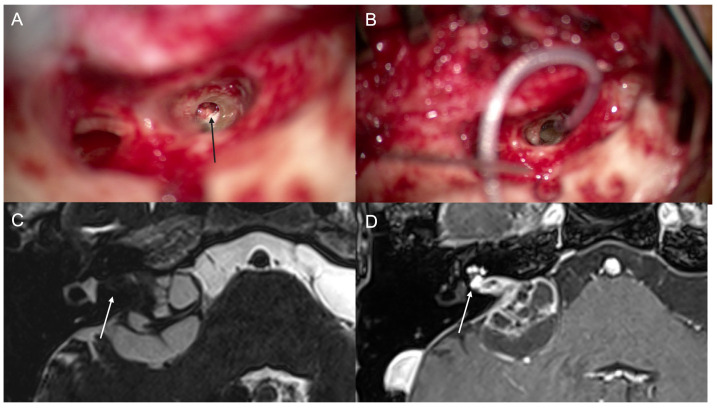
Case #4 (**A**–**D**). During the revision surgery, soft tissue was found in cochlea (black arrow) (**A**). After removal of the tissue, electrode was inserted (**B**). Postoperative T2-weighted MR with magnet removal 2 years later (**C**) shows a mass in IAC (white arrow) and CPA. T1-weighted MR with gadolinium enhancement shows enhanced mass in IAC and cochlea (white arrow) (**D**).

A 30-year-old male reported bilateral progressive hearing loss, lasting 5 years on the left side and 2 years on the right side. Brain MR imaging revealed a left vestibular schwannoma, leading to the patient undergoing mass excision via a suboccipital approach by a neurosurgeon. Two years post-surgery, the patient experienced sudden sensorineural hearing loss on the right side. The patient received high-dose systemic corticosteroids and intratympanic steroid injections, but there was no improvement in hearing. Subsequently, the patient underwent CI on the right side. After 7 years of using the CI, the patient suddenly lost its benefits for an unknown reason. The aided SDT on the right side was 34 dB, and WRS was 4% at 65 dB. Revision CI surgery was performed. During the revision CI, granulation-like tissue, later identified as schwannoma, was found and removed (Figure 4). Genetic testing revealed a mutation in the NF-2 gene. Three months after the revision CI, the right side’s aided PTA was 60 dB, and the WRS was 0% at 65 dB. The CI device used was the synchrony + FLEX 24 from MED-EL. Postoperatively, the mass was evaluated via MR after removal of the magnet (Figure 4) and there was a mass in the IAC and CPA. 

## 4. Discussion

ILS is characterized by non-specific symptoms, often leading to a delayed diagnosis due to its slow growth pattern. The management approach to ILS depends on factors such as tumor size, location, and the patient’s symptoms [5] since the case report shows the simultaneous removal of the mass and CI in patients with ILS [15].

Surgical removal and CI insertion are excellent ways to restore hearing. Carlson et al. reported 10 cases of simultaneous CI electrode insertion and mass removal. Postoperative results varied: 0–88% of WRS [9]. Plontke et al. also reported eight cases of CI electrode insertion with mass removal. At the three-month postoperative follow-up, seven out of eight patients showed a 45–75% WRS, which improved at the last follow-up [10]. Plontke et al. also reported a good postoperative word recognition from 16 patients with intracochlear or intravestibular schwannoma. After simultaneous removal of the mass and the insertion of a CI electrode, a high WRS was reported (75%) even in cases with partial cochleosectomy. [16]. Haussler et al. also reported the results of simultaneously removing the intracochlear mass and CI electrode from 10 patients. The maximum Freiberg monosyllable test results until a 24-month follow-up ranged from 0% to 50% [12]. Ha et al. reported four cases of intracochlear schwannoma, showing a 15–60% WRS at the 6-month follow-up [17].

Like previous reports, simultaneous CI with mass removal appears to be a safe and effective treatment for restoring hearing. However, the postoperative audiological results vary. The patients included in our report also exhibited variable postoperative audiological outcomes. Similar to the report by Carlson et al. [9], two of our patients had bilateral vestibular schwannomas extending to the cerebellopontine angle, leading to poor postoperative audiological outcomes.

This study included two patients with NF2, a condition associated with bilateral vestibular schwannomas. Over the years, following the first CI in an NF2 patient in 1989, hearing rehabilitation for NF2 patients has shown significant results. Although early CI outcomes in NF2 patients were initially disappointing compared to those in the general population, there is now promising potential for improved hearing outcomes within this specific group [18].

A study by Lustig et al. examined the outcomes of CI in NF2 recipients, revealing the heterogeneity of the results. These were mainly influenced by the presence or absence of contralateral hearing [18]. These findings underscore the importance of considering the unique challenges faced by NF2 patients when contemplating CI use. In this report, patients with bilateral vestibular schwannomas exhibited a 0% WRS postoperatively following CI. Achieving satisfactory audiological outcomes for patients with NF2 is very challenging, in which case an Auditory Brainstem Implant (ABI) may be considered.

Patient 1 underwent prior stereotactic radiosurgery (SRS) followed by CI. The literature on radiosurgery treatment for ILS is limited. Campos Paiva et al. reported a successful case of transmodiolar ILS treated with Gamma Knife, demonstrating positive outcomes regarding improved quality of life and preserved hearing post-treatment [19].

However, it is important to note that SRS for ILS carries a higher risk of hearing loss compared to VS due to the tumor’s proximity to the cochlea. The anticipated damage to neural structures, particularly the cochlear spiral ganglion cells, significantly decreases the likelihood of successful hearing rehabilitation through CI following radiotherapy for ILS [10].

According to this study, an extension of the mass into the IAC/CPA showed worse results than the case with intracochlear mass. Extensions into the CPA could be associated with more damage to the cochlear nerve and contributed to the postoperative audiological success in the cases of a Schwannoma inside the cochlea. In intracochlear tumors, the vestibular function was reported to be maintained after the simultaneous removal and CI. [20]. This implies that the extent of tumor involvement (sparing the IAC or CPA) seems to be important to preserving the other inner ear function.

Following CI, monitoring residual tumors post-surgery becomes challenging due to CI artifacts interfering with MRI scans [21]. Utilizing a Material Artifact Reduction Sequences (MARS) protocol may help to overcome the challenges that CI artifacts pose [22].

## 5. Conclusions

This study emphasizes the significance of conducting CI simultaneously with an excision of the mass inside the cochlea. The findings indicate a specific improvement in hearing for cases of sporadic schwannoma, regardless of the CI type used. However, there was 0% WRS in the two NF2 patients with a mass in the IAC.

## Data Availability

All data generated or analyzed during this study are included in this article. Further enquiries can be directed to the corresponding author.
